# Accessing Methyl Groups in Proteins via ^1^H-detected MAS Solid-state NMR Spectroscopy Employing Random Protonation

**DOI:** 10.1038/s41598-019-52383-3

**Published:** 2019-11-04

**Authors:** Sam Asami, Bernd Reif

**Affiliations:** 1 Munich Center for Integrated Protein Science (CIPS-M) at Department Chemie, Technische Universität München (TUM), Lichtenbergstr. 4, 85747 Garching, Germany; 2 Helmholtz Zentrum München (HMGU), Deutsches Forschungszentrum für Gesundheit und Umwelt, Institute of Structural Biology (STB), Ingolstädter Landstr. 1, 85764 Neuherberg, Germany

**Keywords:** Biochemistry, Biophysics, Structural biology, Chemistry, Biophysical methods, NMR spectroscopy

## Abstract

We recently introduced RAP (reduced adjoining protonation) labelling as an easy to implement and cost-effective strategy to yield selectively methyl protonated protein samples. We show here that even though the amount of H_2_O employed in the bacterial growth medium is rather low, the intensities obtained in MAS solid-state NMR ^1^H,^13^C correlation spectra are comparable to spectra obtained for samples in which α-ketoisovalerate was employed as precursor. In addition to correlations for Leu and Val residues, RAP labelled samples yield also resonances for all methyl containing side chains. The labelling scheme has been employed to quantify order parameters, together with the respective asymmetry parameters. We obtain a very good correlation between the order parameters measured using a GlcRAP (glucose carbon source) and a α-ketoisovalerate labelled sample. The labelling scheme holds the potential to be very useful for the collection of long-range distance restraints among side chain atoms. Experiments are demonstrated using RAP and α-ketoisovalerate labelled samples of the α-spectrin SH3 domain, and are applied to fibrils formed from the Alzheimer’s disease Aβ_1-40_ peptide.

## Introduction

Methyl groups are valuable probes for structure and dynamics investigations of proteins and possess favorable relaxation properties, such as short *T*_1_ and long *T*_2_ times in deuterated microcrystalline protein samples. An efficient labelling scheme for selective Ileδ1 and Valγ1,2/Leuδ1,2 methyl labelling was introduced for solution-state^1–5^. In the solid-state, first pyruvate labelling was employed to yield high-resolution ^1^H,^13^C correlation spectra^6^. Pyruvate yields labelling of all methyl containing amino acids. However, as the precursor was commercially only available as 60% deuterated, methyl groups in the protein appeared as mixtures of CH_3_, CH_2_D, CHD_2_ and CD_3_ isotopomers, which compromised the overall sensitivity of the experiment. Later on, α-keto-acid and acetolactate^7^ labelling was employed to yield methyl relaxation rates^8^, side chain order parameters^6^ and methyl-methyl distance restraints^9,10^ in microcrystalline proteins in the solid-state.

We have subsequently observed that high resolution ^1^H,^13^C correlation spectra can be acquired in case the glucose employed in the bacterial growth medium is only 97% deuterated^11^. This triggered the development of RAP (reduced adjoining protonation) labelling schemes in which H_2_O was added to the deuterated minimal medium which contained ^2^H,^13^C glucose^12^. A similar approach (coined fractional deuteration and inverse fractional deuteration) was introduced later by others, in which the minimal medium consists of ^1^H,^13^C glucose and D_2_O, or ^2^H,^13^C glucose and H_2_O, respectively^13,14^. Alternatively, side chain protons can be introduced by addition of protonated ^13^C,^15^N labelled amino acids in the bacterial growth medium, which contains D_2_O and ^2^H,^13^C glucose otherwise. This approach was coined proton cloud labelling^15^. Finally, use of amino acid oxidases allows to generate keto-acids from amino acid mixtures. Transaminases in turn convert the keto-acids in specifically Cα protonated amino acids during bacterial growth^16^.

We compare here methyl order parameters, including dipolar coupling anisotropy values as well as asymmetry parameters, obtained from a selectively ^13^CHD_2_ Leu/Val methyl labelled α-spectrin SH3 sample and a randomly protonated SH3 sample using the RAP labelling scheme^12,17^, respectively. Surprisingly, we find that the intensities of even the 5% RAP labelled sample are rather high, and comparable to the intensities obtained for the α-keto-acid labelled sample. As expected, the order parameters for the two samples are rather similar. The RAP labelled sample, however, yields labelling of all methyl groups with similar enrichment of protons for all methyl bearing side chains. We believe that this labelling scheme will be employed widely in the future as it is easy to implement, allows to obtain assignments via HCCH type experiments^18^, as well as information on local dynamics and structure.

## Results

### ^1^H,^13^C correlation spectroscopy

**Figure 1:**
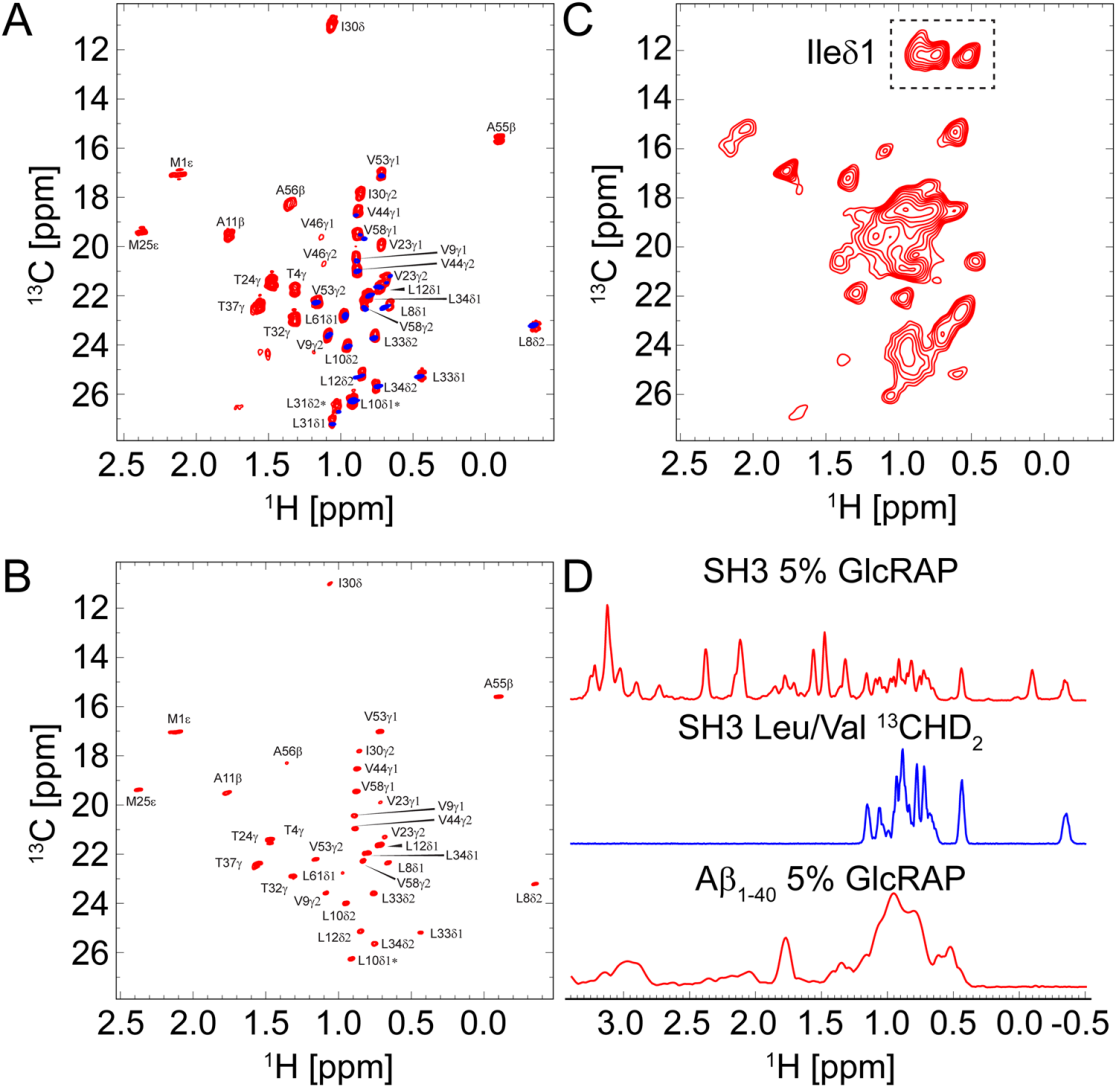
(**A**) 2D ^1^H,^13^C methyl correlation spectra of a 5% GlcRAP and a Leu/Val ^13^CHD_2_ sample of α-spectrin SH3 at an external magnetic field of 14.1 T (600 MHz), adjusting the MAS frequency to 50 kHz and the effective sample temperature to 20–25 °C. All samples were prepared in a 100% D_2_O buffer (details are given in the Methods part). The ^1^H, ^13^C HMQC spectrum of the 5% GlcRAP sample is shown (red) superimposed with the respective spectrum of the Leu/Val sample (blue). The ^13^C line width of the Leu/Val sample amounts to 25 Hz. The ^1^H line width for both samples is on the order of ~22–25 Hz. The methyl cross peaks in the ^13^C dimension of the 5% GlcRAP spectrum are split into doublets due to evolution of the ^1^*J*_C,C_ scalar couplings (except for Met). (**B**) ^1^H,^13^C constant-time HSQC spectrum of the 5% GlcRAP sample using a constant-time delay $T=1/{J}_{C,C}={\rm{28.6}}\,{\rm{ms}}$. The ^13^C line width is ~25 Hz (or ~16 Hz if the indirect evolution period is doubled using mirror-image linear prediction). The apparent ^13^C methyl *T*_2_ time for both samples is roughly around ~30 ms. (**C**) ^1^H,^13^C HMQC spectrum of 5% GlcRAP labelled Aβ_1-40_ fibrils, recorded at an external magnetic field of 16.4 T (700 MHz) and a MAS frequency of 18 kHz. The ^1^H line width is on the order of ~130 Hz. (**D**) 1D projection of 2D ^1^H,^13^C HMQC spectra of all samples. All pulse sequences are given in Fig. S1. The figure was generated with Adobe Illustrator CS5 V.15.02.

We recorded ^1^H-detected 2D ^1^H,^13^C HMQC spectra for the SH3 domain of α-spectrin comparing two different labelling schemes, namely 5% GlcRAP labelling^17^ (red) and selective Leu/Val ^13^CHD_2_ methyl labelling^3^ (blue, Fig. 1A). Both samples were crystallized in a 100% D_2_O buffer (details are given in the Methods part). We achieved high resolution for both samples spun at a MAS frequency of 50 kHz yielding ^1^H line widths on the order of ~22–25 Hz. Clearly, one major benefit of the 5% GlcRAP labelling is that all methyl groups (Ala, Ile, Leu, Met, Thr, Val) become observable at once with comparable resolution, while in the Leu/Val labelling only two out of six methyl-bearing amino acids can be detected (Fig. 1D).

Since the 5% GlcRAP sample was uniformly ^13^C labelled, evolution of ^13^C,^13^C scalar couplings affects the resolution in the methyl region, except for methionine methyl groups, which lack an adjacent carbon (Fig. 1A). In the Leu/Val sample only one carbon was isotopically enriched, which improved the methyl resolution in the indirect ^13^C dimension. However, appearance of splittings in the ^13^C dimension can be avoided by performing a constant-time HSQC experiment (Fig. S1B) at the expense of sensitivity. As shown in Fig. 1B, the application of a constant time sequence yields the same resolution for the 5% GlcRAP sample as for the Leu/Val sample. The ^13^C line widths are about 25 Hz for both samples. In a constant time experiment, magnetization does not decay as a function of the *t*_1_ evolution period, which is beneficial for mirror-image linear prediction^19,20^. Doubling the indirect *t*_1_ evolution period by linear prediction yielded a further improvement of the ^13^C resolution. The ^13^C line width thus amounts to ~16 Hz for the 5% GlcRAP sample. As shown previously^21^, ^13^C,^13^C splittings can be chemically removed using the [2]-GlyRAP or [1,3]-GlyRAP instead of the GlcRAP labelling scheme, respectively, since the former utilizes [2]- or [1,3]-^13^C glyercol instead of glucose as the carbon source during protein expression^22^. With this type of labelling, constant-time pulse sequence elements can be circumvented, which significantly enhances sensitivity as real time experiments can be used and at the same time yields high resolution comparable to Leu/Val labelling.

We applied the 5% GlcRAP labelling scheme, furthermore, to amyloid fibrils, formed by the Alzheimer’s Aβ_1-40_ peptide^23–25^. We recorded 2D ^1^H,^13^C HMQC spectra for a 5% GlcRAP labelled Aβ_1-40_ fibril sample as shown in Fig. 1C. Even though the resolution in the ^1^H dimension is less compared to spectra of the microcrystalline SH3 domain, which is presumably due to the low spinning frequency of 18 kHz, fibril polymorphism and lower sample homogeneity, there is still a good spectral dispersion of the methyl groups. Solid-state NMR investigations of Aβ fibrils usually rely on ^13^C spectroscopy^26,27^. However, using the ^13^C chemical shift information of the methyl group does not allow to disperse the Ileδ1 signals, whereas the methyl groups appear resolved in the ^1^H dimension (Fig. 1C). We note, that Aβ_1-40_ contains only two isoleucine residues, while clearly three Ileδ1 resonances were detected, revealing the polymorphic nature of the fibril. In addition, the spectral sensitivity is significantly enhanced by ^1^H detection.

### 5% GlcRAP versus selective Leu/Val methyl labelling

**Figure 2:**
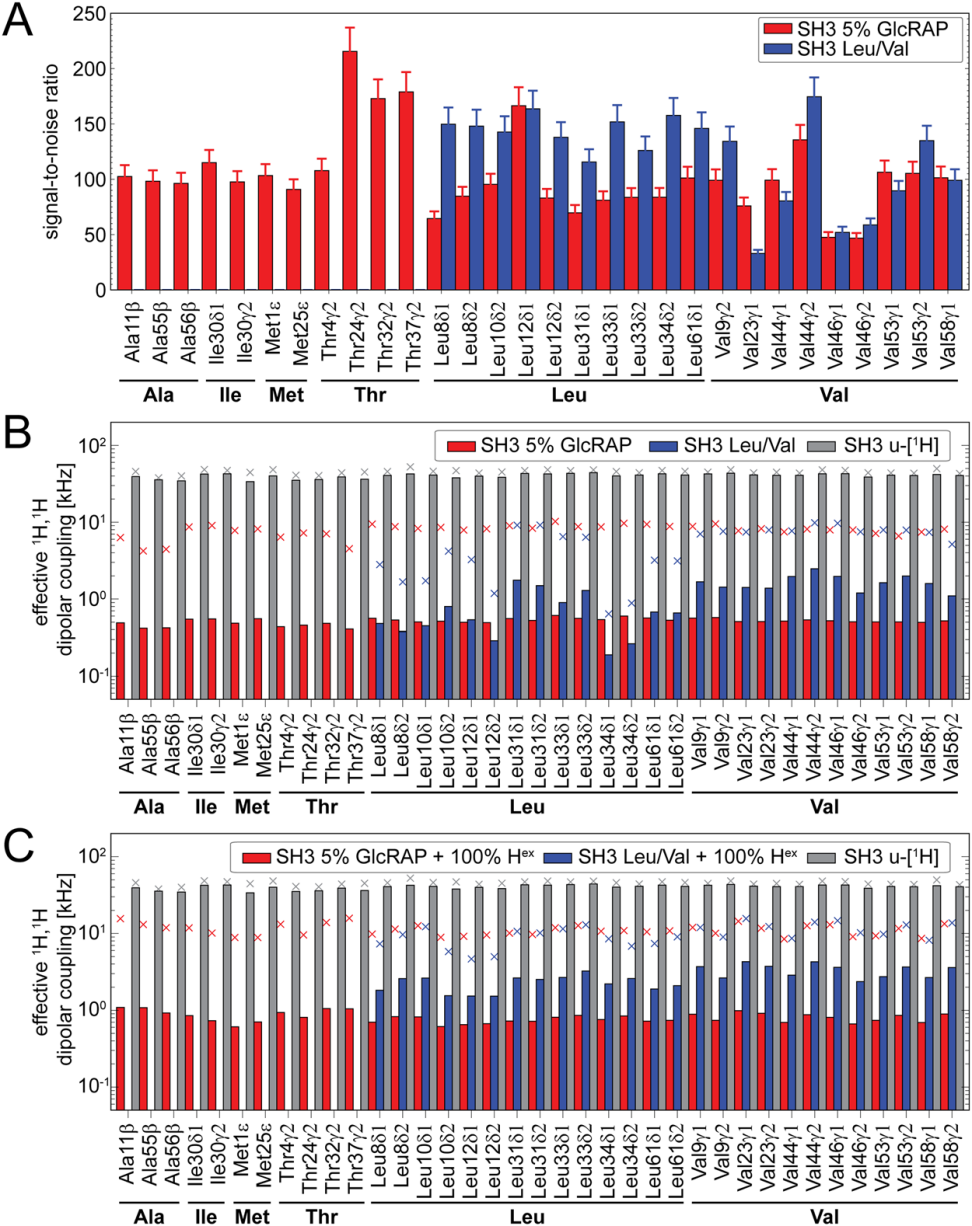
(**A**) Experimental signal-to-noise ratios extracted from 2D ^1^H,^13^C HMQC spectra of 5% GlcRAP (red) and selectively Leu/Val ^13^CHD_2_ labelled α-spectrin SH3 (blue). The experiments were run under comparable conditions. The experimental time to record a spectrum for each sample was ~40 min (details are given in the Methods part). (**B**) *In silico* calculated ^1^H,^1^H effective dipolar couplings for methyl groups based on the 1 μs MD relaxed crystal structure of the SH3 domain (note the logarithmic y-scale). Calculations were carried out for the proton spin diluted structures according to the 5% GlcRAP labelling scheme (red bars), the Leu/Val ^13^CHD_2_ labelling scheme (blue) and for the uniformly protonated structure (grey). Crosses depict the upper 2σ confidence interval. (**C**) The same as (**B**) incorporating all exchangeable protons. The figure was generated with Adobe Illustrator CS5 V.15.02.

In the following, we compared signal-to-noise ratios for the 5% GlcRAP and the Leu/Val labelled SH3 domain obtained in 2D ^1^H,^13^C HMQC experiments (Fig. 2A). Overall, the gain in sensitivity for Leu/Val labelling over 5% GlcRAP is up to a factor of ~3. We further compared the sensitivity to the proton concentration at a given methyl site for the respective labelling scheme. The average proton concentration at a methyl site in a Leu/Val ^13^CHD_2_, a 5% GlcRAP and an uniformly protonated sample is 16.7% (*p* = (1/3) × (1/2)), ~3.5% (averaged) and 100%, respectively. The proton incorporation rate for 5% GlcRAP has been experimentally estimated previously by solution-state NMR^12^. Due to the higher concentrations of protons in Leu/Val samples, we, thus, expect a signal-to-noise gain of a factor of ~5 over the 5% GlcRAP labelled sample. The lower than expected gain can be caused by several factors: (i) differences in the ^1^H dipolar network causing dipole-mediated line broadening, (ii) sample inhomogeneity, (iii) varying degrees of H_2_O impurities, (iv) different amounts of protein in the two rotors, (v) low incorporation of α-keto-acid precursors into the protein and (vi) pH differences.

The latter is expected to only have a small effect on the sensitivity of methyl groups as compared to the strongly pH-dependent exchangeable protons, such as amides. On the other hand, the sample homogeneity has a severe impact on sensitivity, however, we obtained similar apparent linewidths for both samples. Varying H_2_O-concentrations of the crystal solvent among both samples correlates to the protonation state at exchangeable sites and can, in principle, add to the ^1^H dipolar network. Both samples were lyophilized two times in 100% D_2_O, yet their H_2_O contents slightly varied due to different storage times. Based on 1D ^1^H spectra, we estimate the amide concentration to be around ~3% (~6%) for the Leu/Val (5% GlcRAP) sample at the time of measurement. A higher concentration of exchangeable protons can reduce the *T*_1_ relaxation time of amides by about ~10%^28^ and to a smaller extent also of methyl groups, which would increase the signal-to-noise ratio per unit time in favor of the 5% GlcRAP sample. We packed both rotors using the same ultracentrifuge device and, on the basis of previous experience^29^, we estimate the error in the amount of material in both samples to be on the order of 5%. In Fig. 2A, we assumed error bars of 10%. To assess the incorporation level of the α-keto-acid precursor molecule into the Leu/Val labelled SH3 protein, we compared the experimental to the theoretical mass (Fig. S2). Both masses agree well, which indicates a full incorporation of the precursor molecule.

We further investigated the ^1^H dipolar coupling network emerging from both labelling schemes. We estimated, therefore, the effective dipolar coupling based on the crystal structure of the SH3 domain for both schemes as well as for an uniformly protonated sample (details given in the Methods part). For an approximate validation of our models, we compared the theoretical mass calculated for the ensemble of the *in silico* structures to the experimentally measured protein mass and obtained a high correlation with a very small bias towards larger theoretical masses (Fig. S2). This indicates that in the measured sample the proton concentration was slightly higher than in the model due to experimental errors and unaccounted isotope impurities throughout the protein expression procedure, respectively. A deviation from the expected proton concentration within both samples will, of course, also contribute to some extent to the observed signal-to-noise differences (Fig. 2A).

In Fig. 2B the averaged effective ^1^H,^1^H dipolar coupling is shown, based on *in silico* structures. The mean value is larger for the Leu/Val sample compared to the 5% GlcRAP sample, respectively, and largest for the uniformly protonated sample as expected. We note, that the predominant protonated methyl isotopomer species is ^13^CHD_2_ for both the Leu/Val and 5% GlcRAP sample, respectively^12^. Other methyl isotopomers, which exhibit potentially large intra-methyl ^1^H,^1^H dipolar couplings, namely ^13^CH_2_D and ^13^CH_3_, can, in principle, be also populated in 5% GlcRAP samples, but are ~5 to ~20 fold less frequent than ^13^CHD_2_. Here, we included all possible isotopomers in the *in silico* models based on the experimentally derived statistics for proton incorporation^12^. Even though, the average effective dipolar coupling for the 5% GlcRAP sample is very small, we find large couplings within the 2σ (95.45% confidence) confidence limit. This is because infrequently there are singular structures, which contain higher protonated isotopomers, that add to the variance of the averaged effective dipolar coupling in 5% GlcRAP samples.

For calculation of the effective dipolar coupling we employed a very simple descriptor assuming static structures, which can, in principle, cause a systematic offset of the coupling value. Despite a potential offset, the dipolar couplings are approximately uniform for all methyl sites in the 5% GlcRAP sample due to the underlying random nature of the labelling scheme, whereas for the Leu/Val sample the coupling values strongly fluctuate. For the latter, the dipolar coupling even exceeds the upper confidence limit of the 5% GlcRAP sample for several residues (Fig. 2B, crosses), despite the absence of higher protonated isotopomers. This is mainly due to the spatial bundling of Leu and Val residues in the hydrophobic core region of the protein and a concomitant increase of the local proton density as Leu/Val methyl groups are overall the only proton-bearing residues in this labelling scheme. The situation is clearly worse for larger proteins with extensive, methyl-rich hydrophobic regions. It is worth noting, that probing all methyl groups (Ala, Ile, Leu, Met, Thr, Val) with one sample using selective methyl labelling^30^ would require the addition of further costly precursor molecules during protein expression. This would significantly add to the proton density as shown in Fig. S3, and, by far, exceed the favorable spectroscopic conditions found in 5% GlcRAP samples, which inherently contain all ^1^H-detectable methyl groups.

We further investigated the possibility to combine the 5% GlcRAP and the Leu/Val labelling scheme with full back-substitution of exchangeable protons (Fig. 2C). Evidently, due to the implicit low proton concentration in the 5% GlcRAP sample the effective ^1^H,^1^H dipolar couplings only mildly increase upon 100% proton back-substitution as opposed to the Leu/Val sample, making the former more suitable for proton detection of aliphatic and amide resonances using the same sample.

### Structural restraints

**Figure 3:**
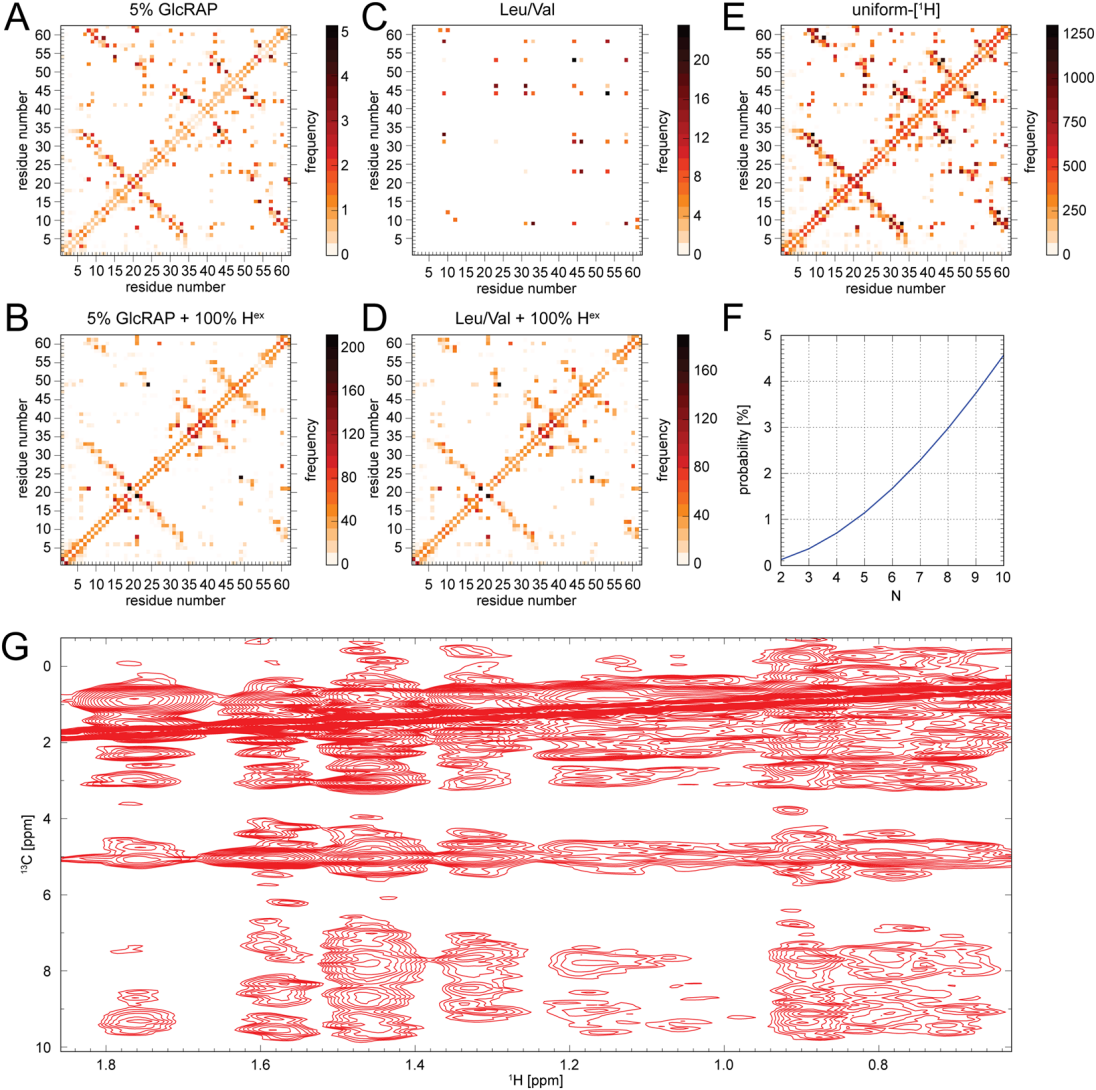
(**A**–**E**) ^1^H,^1^H contact map for the 1 μs MD relaxed crystal structure of the SH3 domain of α-spectrin for an upper ^1^H,^1^H distance limit of 5 Å. The maps were calculated for the (**A**) 5% GlcRAP and (**C**) Leu/Val labelling scheme, respectively, as well as for an (**E**) uniformly protonated sample. Full proton back-substitution was also considered for the former in (**B**) and (**D**). (**F**) The probability *P* to detect at least one ^1^H,^1^H contact for *N* adjoining sites assuming a likelihood of *p* = 0.035 to find a proton at a given site. (**G**) Methyl region of a ^1^H,^1^H 2D projection extracted from a ^1^H-detected 3D H(H)CH RFDR spectrum (τ_mix_ = 8 ms) using the 5% GlcRAP labelled SH3 domain^12^. The spectrum was recorded at an external magnetic field of 14.1 T (600 MHz) and a MAS frequency of 20 kHz. The H_2_O content in the microcrystalline sample was < 10%. Clearly, a number of ^1^H,^1^H contacts were detected including H^met^-H^met^, H^met^-H^ali^, H^met^-solvent and H^met^-H^N^. The figure was generated with Adobe Illustrator CS5 V.15.02.

In the following, we compared the 5% GlcRAP and Leu/Val labelling in terms of accessibility of long-range structural restraints, which are crucial for biomacromolecular structure determination. Using *in silico* models of the SH3 domain (*vide supra*), we calculated the frequency of encountering ^1^H,^1^H pairs within 5 Å for both labelling schemes as well for an uniformly protonated sample without and with full backsubstitution of exchangeable protons (Fig. 3A–E). We find, that the 5% GlcRAP labelled sample (Fig. 3A) shows essentially the same pattern of ^1^H,^1^H contacts as compared to the uniformly protonated sample (Fig. 3E) despite the considerably lower proton concentration, unlike the Leu/Val labelled sample, which only shows a very limited number of contacts (Fig. 3C), even though at a higher frequency compared to the 5% GlcRAP labelled sample. The 5% GlcRAP labelled sample additionally allows to sequentially connect residues as indicated by the contacts in parallel to the diagonal (Fig. 3A).

Including all exchangeable protons significantly elevates the frequency of ^1^H,^1^H contacts for the 5% GlcRAP sample (Fig. 3B), which drastically increases the probability to observe cross peaks that could serve as restraints to interconnect proximal residues. Similarly, for the Leu/Val sample the frequency of finding interconnecting ^1^H,^1^H contacts substantially increases (Fig. 3D) and resembles the 5% GlcRAP contact map. We note, however, that the Leu/Val sample with full proton backsubstitution shows considerably larger ^1^H,^1^H dipolar couplings compared to the 5% GlcRAP sample (Fig. 2C), indicating spectroscopic benefits for the latter.

The low proton concentration in the 5% GlcRAP sample affects the measurement of structural restraints that rely on the observation of ^1^H,^1^H contacts (Fig. 3A). To first order, with *p* being the likelihood of finding a proton at a given site yields a probability *P* = *p*^2^ to observe a cross peak for two structurally proximal protons. This probability *P* is vanishing if *p* is small as for methyl groups in 5% GlcRAP labelled samples (*p* ≈ 0.035). However, if we consider multiple proximal proton sites for a particular molecule within the ensemble, then the likelihood to find at least one cross peak among those sites increases beyond *p*^2^. For *N* proximal proton sites, there are ${2}^{N}-N-1$ cross peak combinations. The probability *P* to find a cross peak between one or more pairs is given by $$P={\sum }_{i=2}^{N}(\begin{array}{c}N\\ i\end{array}){p}^{i}{(1-p)}^{N-i}$$ (Fig. 3F).

To experimentally validate the *in silico* results, we recorded a ^1^H-detected 3D H(H)CH RFDR spectrum using the 5% GlcRAP labelled SH3 domain^12^. The ^1^H,^1^H 2D projection is shown in Fig. 3G. Evidently, a large number of ^1^H,^1^H contacts is observable, including H^met^-H^met^, H^met^-H^ali^, H^met^-solvent and H^met^-H^N^, respectively. Furthermore, due to the low proton concentration per molecule, dipolar truncation is less pronounced in the 5% GlcRAP labelled sample, which allows the measurement of long ^1^H,^1^H distances, even up to 9 Å^12^.

### Methyl dipole tensor

So far, we showed that the 5% GlcRAP labelling scheme produces samples, that yield sufficient signal-to-noise in ^1^H-detected solid-state NMR experiments and enables the detection of all methyl groups (Ala, Ile, Leu, Met, Thr, Val), unlike for the selectively methyl-labelled Leu/Val sample (only Leu, Val). Additionally, structural restraints can be obtained (*vide supra*). In the following, we determine dipolar order parameters and motional dependent tensor asymmetries employing REDOR experiments^9^ (Fig. S1C) for all methyl groups in α-spectrin SH3 using the 5% GlcRAP sample (Alaβ, Ileγ2,δ1, Metε, Thrγ2, Leuδ1,δ2, Valγ1,γ2), and for Leuδ1,δ2 and Valγ1,γ2 using the Leu/Val sample, respectively.

The REDOR experiment was shown to be a very powerful approach as it combines ^1^H-detection and accurate determination of the dipolar tensor with little sensitivity towards rf inhomogeneities and mismatches, offset effects and variations of the CSA properties of the involved nuclei^31–34^. Only the presence of remote protons can be an obstacle as REDOR is known to recouple the ^1^H,^1^H homonuclear dipolar coupling^35,36^. However, fast spinning frequencies ≥50 kHz renders the experiment essentially insensitive to remote protons, when applied to perdeuterated and amide back-exchanged proteins^37^. We note, that the 5% GlcRAP labelling scheme yields incorporation of a comparably small concentration of protons into the protein together with an approximately uniform distribution of ^1^H,^1^H effective dipolar couplings throughout the protein (Fig. 2B). This fact reduces systematic errors in quantitative experiments that are sensitive to remote protons, such as the REDOR experiment employed here. On the other hand, for Leu/Val samples the effective dipolar coupling varies significantly as a function of the methyl position. In extreme cases, different proton densities for each methyl group might have to be taken into account in the numerical simulations, which can complicate the data analysis and additionally requires a high-resolution structure of the system under study.

**Figure 4:**
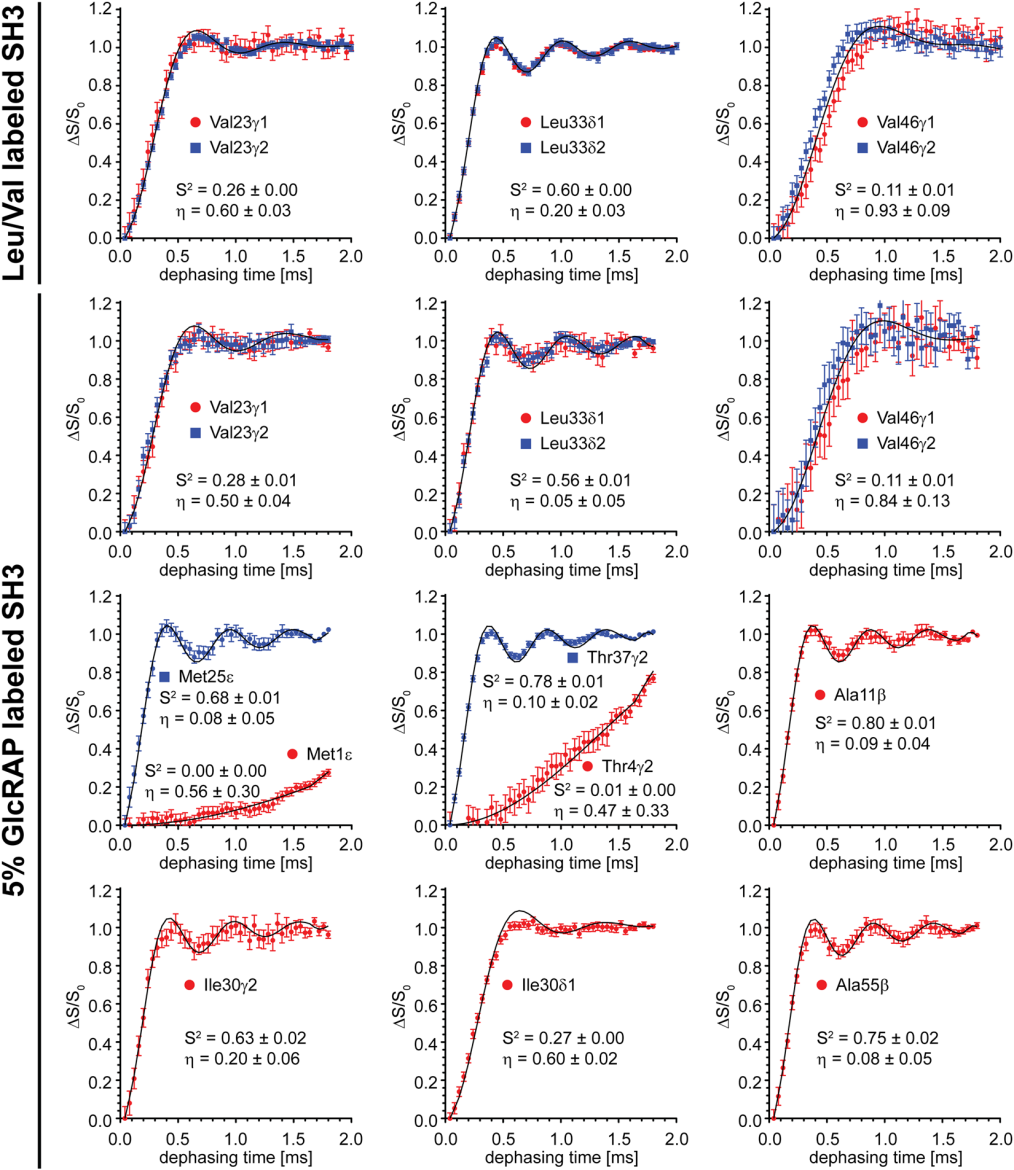
^1^H-detected REDOR dephasing curves, using a selectively Leu/Val ^13^CHD_2_ methyl labelled and a 5% GlcRAP labelled sample of α-spectrin SH3. The external magnetic field was 14.1 T (600 MHz). The effective temperature of the sample was adjusted to 20–25 °C. The MAS frequency was set to 50 kHz. In the experiment, a ζ-delay of 4 µs was employed. The methyl order parameter *S*^2^ (*S*^2^_axis_ in Eq. (4)) and the asymmetry η were determined as described in the Methods section. The values are given at the bottom of each plot. In the first two rows, the dephasing curves were fitted by simultaneously minimizing χ^2^ for both methyl sites (γ1/γ2 for Val and δ1/δ2 for Leu) to improve the fitting convergence. This yielded one order and asymmetry parameter for each Val and Leu residue. However, individual fits for γ1/γ2 (Val) and δ1/δ2 (Leu) were plotted in red and blue to indicate that *pro*R and *pro*S methyl groups show very similar dynamics. The figure was generated with Adobe Illustrator CS5 V.15.02.

Experimental REDOR dephasing curves are shown in Fig. 4 for the Leu/Val and 5% GlcRAP sample, respectively. The dephasing curves for the common Leuδ1,δ2 and Valγ1,γ2 residues show very similar trends for both samples. However, the additional methyl order parameters for Ala, Ileγ2,δ1, Met and Thr, which became accessible using the 5% GlcRAP sample, allow to probe the amplitude of motion in nearly all protein regions. Ala11β displayed the largest squared order parameter (*S*^2^ = 0.80) among all methyls and reflects the dynamics of the protein backbone.

The most significant asymmetric dynamics was detected for Val23, Ile30 and Val46, respectively. The dynamic property of Val23 with η = 0.60 ± 0.03 was also observed by ^2^H line shape analysis reporting the same asymmetry parameter (η = 0.59 ± 0.01)^38,39^. However, the methyl groups of Val46 could not be analyzed before by ^2^H experiments as they are too mobile to yield sufficient sensitivity during the ^2^H,^13^C cross polarization magnetization transfer. For Ile30δ1 a very low order parameter was obtained (*S*^2^ = 0.27), while the dipolar coupling asymmetry was very high (η = 0.60 ± 0.02). Interestingly, the dephasing curve could not be fitted reasonably well by simply assuming an asymmetric tensor, similar to results obtained for ubiquitin^9^. This hints to the presence of a more complex motion on a microsecond timescale. In principle, the dephasing curve could be fitted adequately by assuming contributions from multiple dipolar coupling tensors with different anisotropies and asymmetries.

Furthermore, dipolar order parameters were obtained for the N-terminal residues Met1ε and Thr4γ2. These residues are not visible in the X-ray structures of α-spectrin SH3 due to dynamical disorder, which is also indicated by very low squared order parameters of 0.00–0.01 (Fig. 4). Residues with such low order parameters typically evade detection in cross-polarization experiments under MAS due to very low magnetization transfer efficiencies^40–42^. However, the significant downscaling of the dipolar coupling enhances the coherence lifetime and gives rise to cross-peaks using the INEPT magnetization scheme, which is based on scalar couplings. Similarly, the ^1^H,^15^N backbone dipolar tensors of the highly dynamic N-terminal residues Thr4 and Gly5 were detectable in the perdeuterated and proton back-exchanged SH3 domain using INEPT-based REDOR experiments^37^.

**Figure 5:**
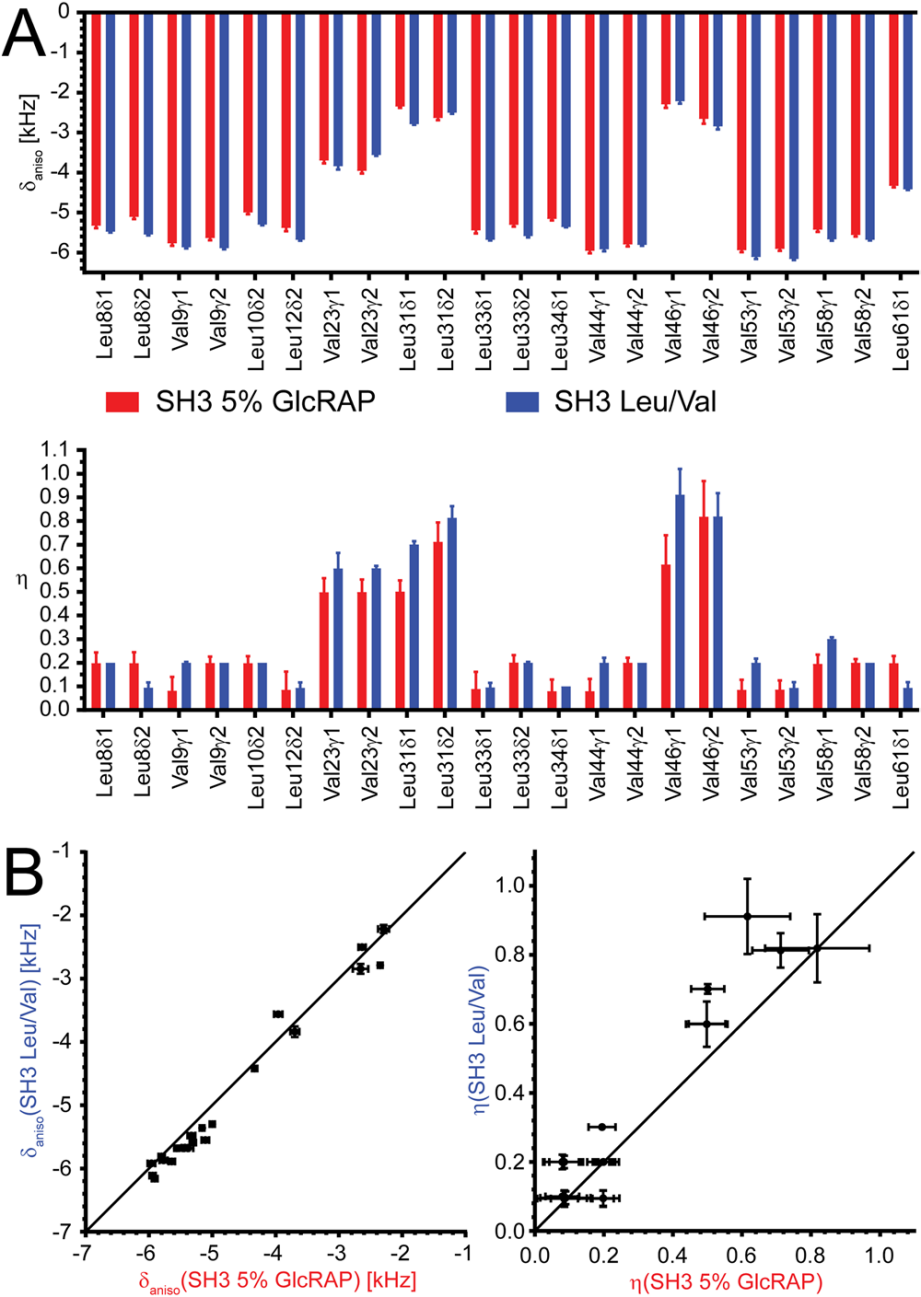
(**A**) Dipolar coupling anisotropies δ_aniso_ (top) and asymmetries η (bottom), using the 5% GlcRAP (red) and the selectively Leu/Val ^13^CHD_2_ methyl labelled sample (blue) of α-spectrin SH3, respectively. All values were determined by fitting REDOR dephasing curves represented in Fig. 4. (**B**) The anisotropies and asymmetries of both samples are linearly correlated, setting the slope to 1.0 and the y-intercept to 0.0. The linear correlation coefficient *R*^2^ is 0.96 and 0.85 for δ_aniso_ and η, respectively. The figure was generated with Adobe Illustrator CS5 V.15.02.

The fitted dipolar coupling anisotropies and asymmetries for Leu and Val methyl groups of both samples are summarized in Fig. 5. The linear correlation coefficients are 0.96 and 0.85 for the anisotropies and asymmetries, respectively. This reveals that both parameters are highly correlated if the two samples are compared, despite the different distribution of remote protons (Fig. 2B), which in turn indicates that the residual ^1^H,^1^H dipolar couplings sensed by the methyl protons in both samples sufficiently averages at a MAS frequency of 50 kHz. However, we note, that for the majority of methyl groups the dipolar coupling anisotropies found in the Leu/Val sample are systematically larger compared to the 5% GlcRAP sample (Fig. 5B). For the former, this indicates a stronger interference of extraneous protons with the REDOR dipolar coupling measurement, which is an effect previously described by others^34^. This is also in line with the *in silico* data (Fig. 2B), which shows larger ^1^H,^1^H dipolar couplings for the Leu/Val sample.

## Discussion

In this work, we studied two different types of labelling schemes and samples to yield high resolution methyl ^1^H,^13^C correlation spectra in the solid-state. The experiments are applied to the microcrystalline SH3 domain of α-spectrin and disease-related amyloid fibrils of the Aβ_1-40_ peptide, respectively. We focus in particular on selective ^13^CHD_2_ methyl labelling of Leu and Val residues using α-keto-acid precursors and 5% GlcRAP labelling. In the first, either one of the two *pro*R or *pro*S methyl groups per residue per molecule is ^13^CHD_2_ labelled, while in the second, all methyl groups are stochastically protonated. The degree of protonation can be adjusted over the amount of H_2_O in the M9 minimal expression medium^12^. Here, we used an expression medium containing 5% H_2_O and 95% D_2_O, respectively, yielding an average methyl proton concentration of about ~3.5%. On the other hand, the proton concentration at either one of the methyl groups in the selectively Leu/Val ^13^CHD_2_ labelled sample is 16.7%.

Despite the low occurrence of methyl protons in the 5% GlcRAP sample, we were able to record high-sensitivity and high-resolution 2D ^1^H,^13^C methyl group correlations, when combining fast MAS frequencies and ^1^H-detection. This is particularly critical for the investigation of biological systems, for which the sample amount is generally limited, irrespective of the sample type, which can comprise of microcrystalls, aggregates or fibrils, such as the herein studied amyloid fibrils of the Aβ_1-40_ peptide. The recently reported structural models of Aβ determined by solid-state NMR spectroscopy relied on ^13^C detection, which suffers from low sensitivity^43,44^. Here, we show that ^1^H,^13^C correlation of Aβ_1-40_ fibrils can be measured with high sensitivity, even at very low MAS frequencies <20 kHz, owing to the largely deuterated background in the 5% GlcRAP sample and ^1^H detection. Moreover, the addition of the highly sensitive ^1^H chemical shift information allows to considerably enhance the resolution and determine structural restraints, despite the low proton concentration.

To first order, the proton concentration correlates with the sensitivity in the ^1^H-detected experiments. However, at the same time, sensitivity is reduced due to enhanced dipole-mediated line broadening^29^, which in turn requires much faster spinning frequencies at the magic angle^45^. It has been shown recently that MAS frequencies beyond 300 kHz are necessary to yield >80% of the theoretical achievable sensitivity in ^13^CH_3_ selectively methyl labelled protein samples. This is way beyond the current technical limit, which is around ~100 kHz. For the GlcRAP labelling, a drastic increase of the proton concentration generally results in significant population of highly protonated methyl isotopomers, namely ^13^CH_2_D and ^13^CH_3_, which not only add to line broadening, but also obscure the spectral resolution due to crowding^29^. Therefore, an optimal GlcRAP sample for detection of methyl groups maintaining sensitivity and resolution can only be achieved at rather low protonation levels. It was shown before, that even using ≥99% D_2_O in the expression medium (corresponding to a 0% GlcRAP sample) yielded detectable proton signals of methyl groups, originating from ≤1% proton impurities of the D_2_O and ≤3% residual protonation of the [^2^H,^13^C] glucose^11^. We find, however, that the optimal trade-off between sensitivity and resolution for methyl correlation spectra is found for ~5% GlcRAP labelling and MAS frequencies ≥50 kHz^29^. In these samples, all the remaining aliphatic sites, such as methine and methylene side chains, backbone and aromatic residues, become protonated and are as well detectable at high resolution using the same sample^12,17^.

One particular benefit of the 5% GlcRAP labelling scheme over the selective methyl labelling scheme is that at once all methyl groups (Alaβ, Ileγ2,δ1, Leuδ1,2, Metε, Thrγ2, Valγ1,2) become accessible in the first at low cost, while in the latter a multitude of precursor molecules has to be employed during expression to achieve full methyl labelling^30^, which, despite the high costs, introduces a significantly higher level of protonation into the sample. On the other hand, having randomly distributed protons in a deuterated matrix, such as in 5% GlcRAP samples, largely reduces the ^1^H,^1^H dipolar network and makes this type of samples particularly attractive for unbiased quantification of dynamics.

We reported previously, that RAP labelling allows to measure structural and dynamical parameters, such as ^1^H,^1^H distance restraints^12^ and aliphatic *R*_1_ relaxation rates^21^, respectively. Furthermore, others determined methyl order and asymmetry parameters for selectively Ileδ1/Leuδ1,δ2/Valγ1,γ2 ^13^CHD_2_ methyl labelled microcrystals of ubiquitin^9^. Here, we report the measurement of methyl dipole tensors of the SH3 domain of α-spectrin by fitting REDOR dephasing curves. As reported recently^34,37^, the REDOR sequence is particularly sensitive to remote protons. Therefore, a 5% GlcRAP labelled sample at fast spinning is optimally suited for this type of NMR experiment. We were able to measure dipolar tensors for all methyl groups with high accuracy. When comparing the order parameters and asymmetries to the Leu/Val sample, we found comparable values within the experimental error, which indicates that at MAS frequencies ≥50 kHz remote protons have very little impact on the REDOR dephasing curve in both samples. However, dipolar coupling anisotropies measured for the 5% GlcRAP sample are slightly smaller compared to the Leu/Val sample, which indicates a weaker interference of remote protons during REDOR measurements for the former. This also reflected by the presented *in silico* data. Moreover, for low spinning rates the effect of remote protons becomes especially challenging for Leu/Val labelled samples as, on average, methyl protons in these samples experience larger ^1^H,^1^H effective dipolar couplings compared to the 5% GlcRAP sample. We further note, that there might be considerable clustering of methyl groups in hydrophobic core regions, depending on the biomolecular system, which can appreciably enhance the effective dipolar coupling in these molecular patches. This is not expected for the 5% GlcRAP labelling scheme due to its intrinsic random nature of protonation.

Using the 5% GlcRAP sample, we were able to determine methyl dipole tensors for all methyl sites, including for Ala, Ileγ2,δ1, Met, Thr. Alanine residues are particularly interesting in terms of dynamics, as the methyl group instantaneously relates to backbone motions, since it is directly connected to the Cα atom^46^. This way, the spectroscopic advantages of methyl groups, such as high sensitivity and resolution, can be exploited to probe the backbone dynamics with improved experimental accuracy and precision.

The dipolar asymmetry parameter contains information about the anisotropy of the side chain motion on the timescale of 1/δ_aniso_^47,48^. Analytical equations to link the methyl order and asymmetry parameters to yield a motional model were given elsewhere^9^. We detected significant asymmetry for Val23 and Val46. The dynamic property of Val23 is also observed by ^2^H line shape analysis, as reported earlier^38,39^. Even though a slightly faster timescale can be probed by ^2^H, we obtained here the same values for the asymmetry parameters. However, the methyl groups of Val46 could not be analyzed by ^2^H experiments, as the methyl groups are too mobile to yield sufficient sensitivity during the ^2^H,^13^C cross polarization magnetization transfer. On the contrary, scalar based transfers were used here for the REDOR experiments, which particularly yield high sensitivity for mobile residues. Val46 is most probably undergoing slow dynamics on the µs time scale since it is not detectable by CP experiments.

In conclusion, the 5% GlcRAP labelling scheme enables ^1^H-detection of all methyl groups as shown for microcrystalline and fibrillar samples, and further allows the determination of structural restraints as well as methyl dipolar order parameters and asymmetries, which are crucial for understanding molecular dynamics. The labelling comes at relatively small cost compared to selectively methyl labelled samples and ensures high resolution and sensitivity, even at slow MAS frequencies <20 kHz. In addition, the 5% GlcRAP labelling scheme can be easily combined with backsubstitution of exchangeable protons with an only mild impact on the ^1^H,^1^H dipolar coupling network.

## Methods

### Sample preparation

The SH3 domain of chicken α-spectrin was produced, as described earlier^49^. Two different labelling schemes were employed in this study, (a) random protonation, following the RAP (reduced adjoining protonation) approach^12,17^, and (b) selective Leu/Val ^13^CHD_2_ methyl labelling^3^. For the RAP sample, hereinafter referred to as 5% GlcRAP, protein expression was carried out using ^15^NH_4_Cl, u-[^2^H, ^13^C] glucose as the sole nitrogen and carbon source, respectively. All buffers used for the M9 medium contained 5% H_2_O and 95% D_2_O.

For the selectively Leu/Val ^13^CHD_2_ methyl labelled sample - referred to as Leu/Val sample - protein expression was carried out using ^15^NH_4_Cl and u-[^2^H, ^12^C] glucose. We added 100 mg/mL of the precursor (two times in 100% D_2_O lyophilized 2-keto-3-methyl-^13^C,d_2_-3-d_1_-4-^13^C,d_2_-butyrate) one 1 hour prior to induction.

Both purified samples were lyophilized two times in 100% D_2_O at pH 3.5 prior to crystallization. Approximately ~3 mg microcrystals were packed into a 1.3 mm rotor by ultracentrifugation (~20 min, ~135.000 × g) employing an ultracentrifuge device^50^. The rotor caps were sealed by gluing, as described earlier^29^. Based on previous experience in packing 1.3 mm MAS rotors using the same setup and device^29^, we estimate the deviation in the amount of material in the rotor to be on the order of 5%. In the analysis presented above, we assumed an error of 10%.

5% GlcRAP labelled Aβ_1-40_ fibrils (with Met at position 0) were produced as described previously, using fibril seeding in a 100% D_2_O buffer^51^. For isotope labelling we followed the same protocol as for the SH3 domain. The final fibrils (~10 mg) were packed into a 3.2 mm rotor using a tabletop centrifuge.

### MALDI-TOF

The labelling efficiency of α-spectrin SH3 samples (5% GlcRAP, Leu/Val and others) were monitored via MALDI-TOF and compared to the expected mass for the respective labelling scheme (*vide infra*). We considered impurities of the uniformly deuterated and ^13^C-enriched carbon source (3% residual protonation and 1% ^12^C). An excellent agreement between the experimental and simulated mass was achieved (Fig. S2).

### NMR spectroscopy

NMR experiments with the SH3 domain of α-spectrin were carried out at 50 kHz MAS using a Bruker BioSpin Avance spectrometer operating at a ^1^H Larmor frequency of 600 MHz (14.09 T), equipped with a commercial 1.3 mm triple-resonance probe. The effective sample temperature was adjusted to ∼20–25 °C using the chemical shift difference between the solvent resonance and Leu8δ2. Experiments with the Aβ_1-40_ peptide were performed at a ^1^H Larmor frequency of 700 MHz (16.4 T) and a MAS frequency of 18 kHz, using a commercial 3.2 mm triple-resonance probe. The employed rf fields on the ^1^H and ^13^C channels for hard pulses were 119 kHz (109 kHz) and 100 kHz (62.5 kHz) at 600 MHz (700 MHz). Low-power ^2^H and ^13^C decoupling of 2.5 kHz was applied, using the WALTZ-16 decoupling scheme^52^.

High-resolution ^1^H,^13^C correlations were obtained for all samples via 2D HMQC spectroscopy and ^2^H decoupling during the ^13^C evolution period (Fig. S1A)^12^. No special care was taken for solvent suppression in these experiments. To spectroscopically eliminate splittings in the indirect ^13^C dimension due to the ^13^C,^13^C one-bond *J*-coupling in the 5% GlcRAP sample, we recorded 2D constant-time HSQC spectra (Fig. S1B)^53^. The spectral resolution in the indirect dimension was further enhanced by mirror-image linear prediction^19^.

^1^H,^13^C REDOR experiments were performed using the pulse sequence depicted in Fig. S1C. The rf fields for ^1^H and ^13^C π pulses were 125 and 100 kHz, respectively, during the REDOR recoupling element. The total REDOR dephasing period was a multiple of the rotor period, *τ*_r_, and equal to $2{\tau }_{r}(n+1)$ with *n* ≥ 0. The REDOR shift ζ defines the timing of the ^1^H π pulse with respect to half of the rotor period, which was set here to 4.0 μs. Following the definition for ε described previously^34^ yielded a value of 0.4 setting ζ equal to 4.0 μs at 50 kHz sample spinning. A *z*-filter delay Δ_zf_ of 5 ms and a recycle delay of 2.1 s (3 s) was employed in experiments using the 5% GlcRAP (Leu/Val) labelled SH3 domain. Reference experiments to account for the signal decay during the recoupling period were scattered evenly (every 0.12 ms) over the total dephasing time of 1.92 ms by setting the rf field of the ^1^H π pulse to 0 Hz. The reference points in between were interpolated from a linear correlation. The bulk ^1^H *T*_1_ for both SH3 samples was ~1.4 s.

### Numerical simulations

All numerical simulations were performed with the software package SIMPSON^54^. For REDOR experiments (Fig. S1C), we simulated recoupling curves (*S*) assuming experimentally applied rf fields for ^1^H and ^13^C π pulses and a ζ delay of 4 μs. We employed a two spin system and generated a matrix of dipolar coupling anisotropies (100 Hz steps) and asymmetries (0.1 steps). The respective REDOR reference curves (*S*_0_) were simulated with zero rf field on the ^1^H channel. The typical REDOR dephasing curve (${\mathfrak{S}}$) was finally determined by ${\mathfrak{S}}=({S}_{0}-S)/{S}_{0}$. Rf inhomogeneities were not taken into account.

### Data analysis

The experimental REDOR spectra were processed in Topspin v3.2 and peak volumes were extracted by box integration, using in-house Python scripts. The experimental error was set to two times the standard deviation of the noise amplitude and uncertainties of the fitting parameters were estimated by 1000 Monte Carlo runs. In the reference experiment *S*_0_, the dipolar coupling was not reintroduced, unlike in the recoupling experiment *S*. The relaxation compensated REDOR dephasing curves were calculated according to $({S}_{0}-S)/{S}_{0}$ similar to the simulations (*vide supra*). We performed a two parameter fit, considering the dipolar coupling anisotropy (δ_aniso_) as well as the asymmetry (η). The experimental curves were fit against a grid, in which χ^2^ was minimized to find the best fit value for both parameters. In the case of Leu and Val residues, both methyl groups, δ1/δ2 and γ1/γ2, were fit simultaneously.

### Determination of the methyl dipolar tensor and order parameter

The heteronuclear ^1^H,^13^C dipolar coupling is a powerful probe for fast dynamical processes on the sub-ms timescale. The dipolar coupling can be expressed in Cartesian coordinates in the principal axis frame by a traceless second rank tensor^9^

1 $${\hat{D}}_{ij}={\delta }_{{\rm{aniso}}}(\begin{array}{ccc}1-\eta  & 0 & 0\\ 0 & 1+\eta  & 0\\ 0 & 0 & -2\end{array})$$

Here, $\eta$ is the asymmetry parameter, and ${\delta }_{{\rm{aniso}}}$ is the dipolar coupling anisotropy, which is defined as (in units of [Hz])

2 $${\delta }_{{\rm{aniso}}}=-\,S\frac{{\mu }_{0}\hslash }{8{\pi }^{2}}\frac{{\gamma }_{i}{\gamma }_{j}}{{r}_{ij}^{3}}$$

where *S* is the order parameter, ${\mu }_{0}$ is the magnetic constant, $\hslash$ is the reduced Planck constant, ${\gamma }_{i}$ and ${\gamma }_{j}$ are the gyromagnetic rations of nuclei *i* and *j* (here ^1^H and ^13^C), respectively, and ${r}_{ij}$ the internuclear distance^9,54–56^. Both the asymmetry parameter and the order parameter can vary between zero and one. In the absence of motion, *S* is equal to one and $\eta$ equal to zero. We employed a rigid-limit ^1^H,^13^C bond length for methyl groups of 1.115 Å yielding an anisotropy of −21794 Hz^57,58^. We calculated the experimentally derived order parameter by dividing the determined dipolar coupling anisotropy by the rigid-limit value.

At room temperature, methyl groups steadily undergo fast rotations around their local threefold axis with correlation times on the picosecond timescale. The amplitude of this fast, axially symmetric rotation is characterized by an order parameter ${S}_{f}^{2}$^58–61^,

3 $${S}_{f}^{2}={\{{P}_{2}(\cos \theta )\}}^{2}={\{\frac{3{\cos }^{2}\theta -1}{2}\}}^{2}$$

yielding ${S}_{f}^{2}\approx 0.111$ for an ideal tetrahedral geometry ($\theta =109.5^\circ$). Ideal tetrahedral geometry was assumed for all methyl-bearing amino acids, noting that methyl groups are usually slightly distorted from ideal geometry^61,62^. Due to the fast timescale of the methyl rotation, which is much shorter than 1/δ_aniso_, the dipolar coupling anisotropy is averaged by this rotational motion. Further averaging is induced by motions of the symmetry axis of the methyl group, which are related to the axis order parameter ${S}_{{\rm{axis}}}^{2}$. Thus, a generalized order parameter ${S}_{{\rm{met}}}^{2}$ for the threefold methyl axis can be defined as

4 $${S}_{{\rm{met}}}^{2}={S}_{f}^{2}\times {S}_{{\rm{axis}}}^{2}\approx 0.111\times {S}_{{\rm{axis}}}^{2}$$

### *In silico* calculation of ^1^H,^1^H dipolar couplings

We used a structural ensemble of 32 monomers of the SH3 domain as a template, which was relaxed by MD simulation for 1 μs^21^, and determined the averaged effective ^1^H,^1^H dipolar coupling experienced by the methyl proton at site *j* in monomer *l* over all proximal protons *k* according to the simple descriptor ${d}_{j}=\frac{1}{N}{\sum }_{l}^{N}\sqrt{{\sum }_{k\ne j}{d}_{jkl}^{2}}$, with *N* as the number of considered monomers throughout the calculation^37,63^. ${d}_{j}$ was set to zero if no proton was found at the specific methyl site. We employed a distance cut-off of 10 Å. Intra-methyl ^1^H,^1^H couplings were scaled by ${P}_{2}(\cos \,\theta )$ with $\theta =90^\circ$ yielding a scaling factor of 0.5^64^.

In the calculations, the labelling scheme was taken into consideration. The population of stochastically incorporated protons in the 5% GlcRAP labelling scheme was based on the experimentally determined proton concentrations^12^, that were scaled to match the experimental mass derived by MALDI (*vide supra*). For the Leu/Val ^13^CHD_2_ labelled sample, we assumed an equal distribution of *pro*-R (50%) and *pro*-S (50%) ^13^CHD_2_ methyl groups, while either one of the two methyl groups at the same residue was considered to be labelled, as constrained by the utilized precursor molecule. Since both samples were re-buffered in 100% D_2_O, all exchangeable protons have been considered as ^2^H. For sufficient statistics, we generated 32000 *in silico* structures and calculated the effective dipolar coupling for each methyl group averaged over all structures. We used the corrected standard deviation of the mean with 1/(*N*-1).

## Supplementary information

Supplementary Information
