# Supplementary material for: Accessing Methyl Groups in Proteins via 1H-detected MAS Solid-state NMR Spectroscopy Employing Random Protonation
Source: Sci Rep. 2019 Nov 4;9:15903. doi: 10.1038/s41598-019-52383-3 (PMC6828780; doi:10.1038/s41598-019-52383-3)
Supplement: Supplementary file 1 — Supplementary Information [file 41598_2019_52383_MOESM1_ESM.pdf]

Supplementary Information

Accessing Methyl Groups in Proteins via ^{1}H-detected MAS Solid-state NMR Spectroscopy Employing Random Protonation

Sam Asami,[a]* Bernd Reif[ab]

[a] Munich Center for Integrated Protein Science (CIPS-M) at Department Chemie, Technische Universität München (TUM), Lichtenbergstr. 4, 85747 Garching, Germany

[b] Helmholtz-Zentrum München (HMGU), Deutsches Forschungszentrum für Gesundheit und Umwelt, Institute of Structural Biology (STB), Ingolstädter Landstr. 1, 85764 Neuherberg, Germany

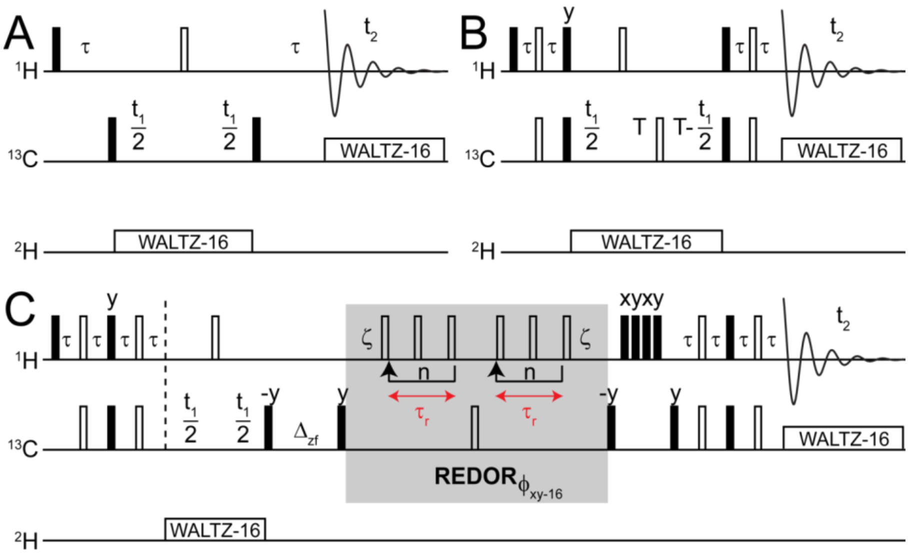

Figure S1. ^{1}H-detected pulse sequences for ^{1}H,^{13}C correlation spectroscopy and determination of ^{1}H,^{13}C dipolar coupling tensors. (A) 2D ^{1}H,^{13}C HMQC pulse sequence with $\tau = 1/2J_{HC} = 3.13$ ms[1]. (B) 2D constant-time HSQC experiment, setting the constant-time delay $T = 1/J_{C,C} = 28.6$ ms and $\tau = 1/4J_{HC} = 1.79$ ms[2]. (C) ^{1}H,^{13}C REDOR pulse sequence[3]. The ^{1}H π pulses during the REDOR period followed the xy-16 phase cycling scheme[4]. The INEPT transfer delay was set to $\tau = 1/4J_{HC} = 1.92$ ms.

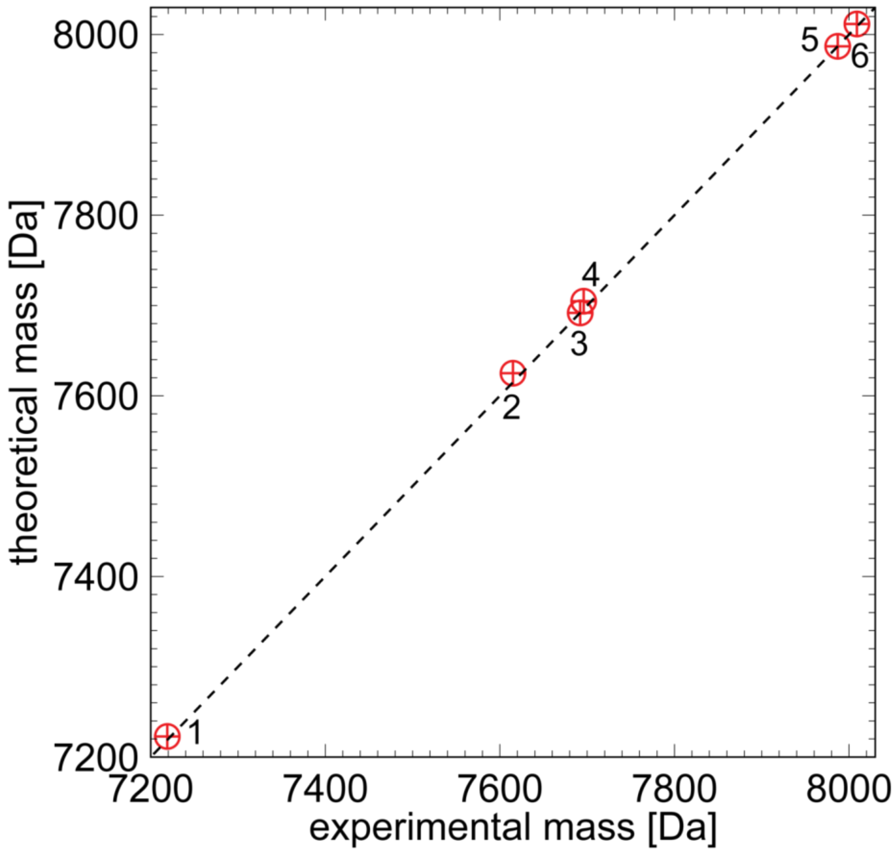

Figure S2. Linear correlation of the experimental to the theoretical mass for differently labelled samples of the SH3 domain of α-spectrin. The samples employed are from left to right: (1) unlabelled, (2) u-[^{1}H,^{13}C,^{15}N], (3) u-[^{2}H,^{15}N], (4) Leu/Val $^{13}CHD_2$ otherwise u-[^{2}H,^{12}C,^{15}N], (5) 5% GlcRAP, (6) u-[^{2}H,^{13}C,^{15}N]. The correlation coefficient R^2 was equal to 0.9998. We yielded an excellent agreement between experimental and theoretical masses, further validating the *in silico* models employed here.

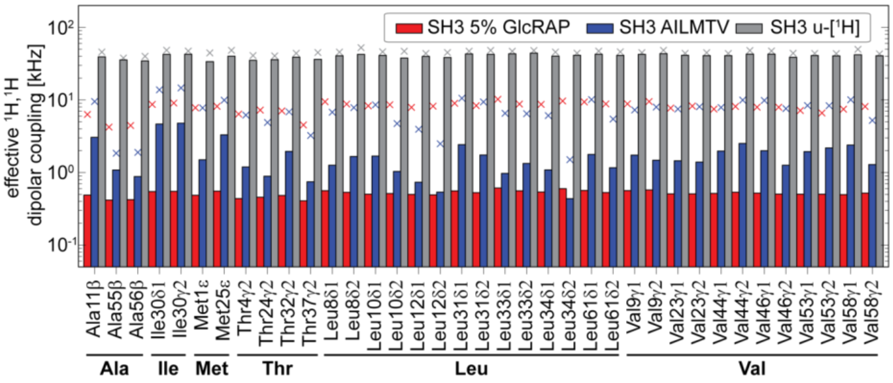

Figure S3. *In silico* calculated ^{1}H,^{1}H effective dipolar coupling for methyl groups based on the 1 μs MD relaxed crystal structure of the SH3 domain of α-spectrin (note the logarithmic y-scale). Here, calculations were carried out for the structures according to the 5% GlcRAP labelling scheme (red bars), the selective Alaβ/Ileγ2,δ1/Metε/Thrγ2/Leuδ1,δ2/Valγ1,γ2 (AILMTV) $^{13}CHD_2$ methyl labelling scheme (blue) and for the uniformly protonated structure (grey). Crosses depict the upper 2σ confidence interval. We note, that for the AILMTV labelling scheme we assumed the $^{13}CHD_2$ isotopomer for all methyl groups. The composition of methyl groups in the SH3 domain is as follows (occurrence is given in parentheses): Ala (3), Ile (1), Leu (7), Met (2), Thr (4), Val (6).

References

1 Asami, S., Schmieder, P. & Reif, B. High Resolution 1H-Detected Solid-State NMR Spectroscopy of Protein Aliphatic Resonances: Access to Tertiary Structure Information. *J. Am. Chem. Soc.* **132**, 15133-15135 (2010).

2 Vuister, G. W. & Bax, A. Resolution Enhancement and Spectral Editing of Uniformly C-13-Enriched Proteins by Homonuclear Broad-Band C-13 Decoupling. *J. Magn. Reson.* **98**, 428-435 (1992).

3 Schanda, P., Huber, M., Boisbouvier, J., Meier, B. H. & Ernst, M. Solid-State NMR Measurements of Asymmetric Dipolar Couplings Provide Insight into Protein Side-Chain Motion. *Angew. Chem., Int. Ed.* **50**, 11005-11009 (2011).

4 Gullion, T. & Schaefer, J. Elimination of Resonance Offset Effects in Rotational-Echo, Double-Resonance Nmr. *J. Magn. Reson.* **92**, 439-442 (1991).
